# Effect of Ca_2_EDTA on Zinc Mediated Inflammation and Neuronal Apoptosis in Hippocampus of an *In Vivo* Mouse Model of Hypobaric Hypoxia

**DOI:** 10.1371/journal.pone.0110253

**Published:** 2014-10-23

**Authors:** Udayabanu Malairaman, Kumaran Dandapani, Anju Katyal

**Affiliations:** Dr.B.R.Ambedkar Center for Biomedical Research, University of Delhi, Delhi, India; Tel-Aviv University, Israel

## Abstract

**Background:**

Calcium overload has been implicated as a critical event in glutamate excitotoxicity associated neurodegeneration. Recently, zinc accumulation and its neurotoxic role similar to calcium has been proposed. Earlier, we reported that free chelatable zinc released during hypobaric hypoxia mediates neuronal damage and memory impairment. The molecular mechanism behind hypobaric hypoxia mediated neuronal damage is obscure. The role of free zinc in such neuropathological condition has not been elucidated. In the present study, we investigated the underlying role of free chelatable zinc in hypobaric hypoxia-induced neuronal inflammation and apoptosis resulting in hippocampal damage.

**Methods:**

Adult male Balb/c mice were exposed to hypobaric hypoxia and treated with saline or Ca_2_EDTA (1.25 mM/kg *i.p*) daily for four days. The effects of Ca_2_EDTA on apoptosis (caspases activity and DNA fragmentation), pro-inflammatory markers (iNOS, TNF-α and COX-2), NADPH oxidase activity, poly(ADP ribose) polymerase (PARP) activity and expressions of Bax, Bcl-2, HIF-1α, metallothionein-3, ZnT-1 and ZIP-6 were examined in the hippocampal region of brain.

**Results:**

Hypobaric hypoxia resulted in increased expression of metallothionein-3 and zinc transporters (ZnT-1 and ZIP-6). Hypobaric hypoxia elicited an oxidative stress and inflammatory response characterized by elevated NADPH oxidase activity and up-regulation of iNOS, COX-2 and TNF-α. Furthermore, hypobaric hypoxia induced HIF-1α protein expression, PARP activation and apoptosis in the hippocampus. Administration of Ca_2_EDTA significantly attenuated the hypobaric hypoxia induced oxidative stress, inflammation and apoptosis in the hippocampus.

**Conclusion:**

We propose that hypobaric hypoxia/reperfusion instigates free chelatable zinc imbalance in brain associated with neuroinflammation and neuronal apoptosis. Therefore, zinc chelating strategies which block zinc mediated neuronal damage linked with cerebral hypoxia and other neurodegenerative conditions can be designed in future.

## Introduction

Hypobaric hypoxia experienced at high altitude has deleterious effects on the Central Nervous System (CNS) mediated through glutamate excitotoxicity, cholinergic dysfunction, oxidative stress and neurodegeneration, [1], [2], [3]. Clinical as well as experimental studies demonstrated the important role played by reactive oxygen species in high altitude hypoxia induced pathological changes in the forebrain region [4], [5]. Reactive oxygen species (ROS) are known to stabilize hypoxia inducible factor-1 α (HIF-1α), a transcription factor which modulates the expression of genes related to hypoxic stress [6], [7], [8], [9]. Further, inducible nitric oxide synthase (iNOS) derived reactive nitrogen species (RNS) and NADPH oxidase derived ROS, have been implicated in inflammation mediated neuronal cell death [10], [11], [12], [13]. Free oxy radicals are known to induce DNA damage resulting in the activation of poly(ADP ribose) polymerase (PARP) [14], a multifaceted enzyme involved in various cytotoxic mechanisms like inflammation, mitochondrial dysfunction, necrosis and apoptosis. Emerging body of evidences suggest apoptosis as the predominant form of neuronal death following cerebral hypoxia [3]. Apoptosis is known to involve cleavage of chromosomal DNA into nucleosomal units by caspases, and activation of Bcl-2 family of proteins in response to apoptotic signals such as cell stress, free radical damage etc. The relative contribution of signaling cascades in mediating neuronal damage hold the key to development of effective therapeutic strategies against hypobaric hypoxia-induced neuronal injury. Glutamate mediated calcium excitoxicity and subsequent neurodegeneration is associated with memory dysfunction on exposure to hypobaric hypoxia [2], [15]. Recently, it has been indicated that zinc, which is co-localized and released along with glutamate, might induce neurophysiological alterations similar to calcium [16]. Further, increased levels of intraneuronal free zinc have been reported in major neurological disorders such as Alzheimer‘s disease, Parkinson‘s disease, amyotrophic lateral sclerosis, stroke, epilepsy, etc. [17], [18], [19]. In our previous study, we reported that hypobaric hypoxia elicited accumulation of free chelatable zinc in the CA3 region of hippocampus accompanied by significant neuronal damage. Chelation of free zinc with Ca_2_EDTA succeed in attenuation of cholinergic dysfunction and neuronal loss associated with memory impairment [20]. Evidences suggest that excess free zinc can be detrimental and exacerbate neuronal damage *in vivo* and *in vitro* [21], [22], [23], [24]. In fact, the zinc chelator Ca_2_EDTA, both *in vivo* and *in vitro*, has been shown to reduce zinc-induced neurotoxicity [25]. Although, the detrimental role of free zinc in a myriad of neurological conditions is well documented, the mechanism of zinc-mediated neuropathogenesis has not been fully illustrated especially in hypobaric hypoxia. Therefore, present study was designed to investigate the effect of Ca_2_EDTA, a specific zinc chelator, on the mechanism underlying hypobaric hypoxia induced neuronal inflammation and apoptosis in Balb/c mice.

## Materials and Methods

### Ethics Statement

The procedures and experimental protocols related to use of Balb/c mice were approved by the Institutional Animal Ethics Committee of Dr. B. R. Ambedkar Center for Biomedical Research, University of Delhi. All efforts were made to minimize animal sufferings and to reduce the number of animals used.

### Animals and Experimental groups

All experiments were performed on adult male Balb/c mice (8–10 wks old, 25–30 g), maintained in animal research facility with 12 h dark/light cycle and 25±2°C temperature at Dr. B. R. Ambedkar Center for Biomedical Research, University of Delhi, Delhi. Animals had free access to food and water *ad libitum*. Balb/c mice were divided into four groups as follows: Normoxia, Normoxia treated with Ca_2_EDTA (1.25 mM/kg in normal saline, *i.p.*), Hypoxia and Hypoxia treated with Ca_2_EDTA.

### Induction of hypobaric hypoxia

Male Balb/c mice were either exposed to hypobaric hypoxia (corresponding to an altitude of 25000 feet for 6 h daily) for 3 days in a specially designed decompression chamber (Model No.SS7001, Seven Stars, India) after gradual adaptation over a period of 1 hr or were kept at room air pressure. Mice had free access to food and water. The rate of ascent to the desired altitude and descent to sea level were at 700 ft/min. The chamber temperature was maintained at 26±2°C.

### Hippocampus homogenate

The total hippocampal homogenate was prepared for immunoblotting and biochemical analysis as described earlier [3]. Briefly, animals were euthanized under excess anesthesia and their brains were harvested to dissect hippocampus [26]. The hippocampus was weighed immediately and homogenized in 10 volumes of cold homogenization buffer (containing 1% IPEGAL CA 630, 10 mM Tris, 150 mM NaCl, 1.5 mM MgCl_2_, 1 mM EDTA, 1 mM EGTA, 1 mM/L bezamidine, 2 µg/mL aprotinin, 10 µg/mL leupeptin and 5 µg/mL pepstatin). The homogenate was centrifuged at 16,000 g for 20 min at 4°C and the supernatant was stored at −80°C until used. Protein concentration was estimated by the method of Bradford et al using spectrophotometer (UV-1601 Shimadzu) at 595 nm.

### Reverse transcriptase PCR amplification

Total RNA was isolated from hippocampus using TRI reagent (Ambion Inc.) and processed for reverse transcription PCR amplification (RT-PCR) as described earlier [3]. The reverse transcription of 5 µg of total RNA was performed using First strand cDNA kit (Fermentas). For polymerase chain reaction 1 µl of cDNA, 1 µl of primers (each 5 pmole), 2 µl of 10× reaction buffer, 0.6 µl of 50 mM MgCl_2_ and 1 unit of DNA polymerase (Biotools, Inc.) was made up to 20 µl with DNase/RNase free water. PCR amplification was carried out by employing a set of specific primers. PCR products were resolved on 1.5% agarose gel and densitometry analysis of PCR products was carried out using Gene Snap software (Gene Tools; Syngene, MD, USA).

### Western blot analysis

Hippocampal homogenate equivalent to 30–35 µg of total protein was denatured with laemmli loading buffer by heating at 95°C for 5 minutes and the samples were resolved on 10–16% SDS-PAGE and electroblotted to nitrocellulose membrane using BioRad semi-dry transblot. The transfer buffer contained in addition, 2 mM CaCl_2_ for metallothionein. Blots were blocked with 3% BSA in PBST over night at 4°C and then incubated with respective primary antibodies - rabbit polyclonal IgG anti- mouse metallothionein 3 (1: 5000); mouse monoclonal IgG anti-mouse Bcl-2, mouse monoclonal IgG anti-mouse Bax (eBiosciences, USA) (1∶500); mouse monoclonal IgG anti-mouse inducible nitric oxide synthase (iNOS) (1: 5000) (Pharmingen, BD Biosciences, USA); anti-mouse GAPDH (1∶5000) (Novus Biologicals, USA) in PBS for 2.5 h at room temperature/overnight at 4°C and then washed thrice with PBS. The blots were then incubated with secondary IgG HRP conjugated goat anti-rabbit (1: 5000) or donkey anti-mouse (1: 5000) antibodies (Santa Cruz Biotechnology, CA, USA) in PBS at room temperature for 2 h. After washing, the blots were developed with 0.06% 3, 3′-diaminobenzidine tetrahydrochloride (DAB), 0.025% cobalt chloride in PBS and 0.01% hydrogen peroxide. The blot images were captured and band density analysis was performed using Gene Snap software (Gene Tools; Syngene, MD, USA). Values of the band density were normalized to the level of respective GAPDH intensity.

### Immunofluorescence

The animals were anesthetized (pentobarbital-sodium 50 mg/kg *i.p.*) and perfused *intracardially* with heparinized (10 U/ml) 0.1 M PBS (pH 7.4) followed by 4% paraformaldehyde in PBS, the whole brain was harvested and stored in a solution of 30% sucrose and 10% glycerol at −80°C until use. Thin sections (5 µm) of the hippocampal region were made using cryotome (CM 1850 Leica, Heidelberg, Germany) as described previously [27] and then processed for TUNEL staining and immunofluorescence studies. Briefly, the sections were permeabilized with 0.1% IPEGAL CA 630 in PBS (pH 7.4) and blocked with 3% BSA in PBS (pH 7.4) for 1 h at 37°C followed by overnight incubation with primary antibodies- rabbit polyclonal IgG anti-mouse metallothionein3 (1: 150); mouse monoclonal IgG anti-mouse iNOS (1∶200); mouse monoclonal IgG anti-mouse COX-2 (1∶150) (Pharmingen, BD Biosciences, USA); rabbit polyclonal IgG anti-mouse HIF-1α (1∶200) and rabbit polyclonal IgG anti-mouse TNF-α (1∶100) (eBioscience, USA). The sections were then washed thrice with PBS, incubated with FITC labeled respective secondary antibody (Santa-cruz biotech) for 1 hour in a dark room and counterstained with Hoechst 33342 (Fluka). The sections were mounted with glycerol/PBS (9∶1), observed under 40× objectives on Nikon Eclipse E600 with Nikon Y-FL Epifluorescence attachment (Tokyo, Japan) and images were acquired with Evolution VF camera followed by analysis with Image-Pro Plus version 5.1.2 software (Mediacybernetics).

### Estimation of Nitric Oxide

The Nitric oxide levels were determined as nitrite plus nitrate as per previous report [3]. The total nitrate from the hippocampal homogenate was reduced to nitrite by using 2% ammonium molybdate and 4% ferrous ammonium sulphate and quantified by using Greiss reagent (1% sulphanilamide, 0.1% naphthylethylenediamine dihydrochloride (NEDA) in 5% orthophosphoric acid) at 540 nm spectrophotometrically.

### Lipid peroxidation

Malondialdehyde (MDA) formation is an indicator to assess oxidative stress induced lipid peroxidation as described by Ohkawa et al, 1979 [28]. Hippocampal homogenate equivalent to 100 µg of protein was mixed with 1 ml of 20% acetic acid (pH 3.5), 1 ml of 0.67% thio barbituric acid and 0.1 ml SDS (8%) and then heated at 100°C for 1 h. The pink chromogen formed in the reaction mixture was extracted with n-butanol and the absorption was measured at 535 nm. The results were expressed as nmol/h/mg protein of MDA formed using a molar extinction coefficient of 1.56×10^5^ M^−1^cm^−1^.

### NADPH oxidase activity

The fluorogenic oxidation of dihydroethidium (DHE) to ethidium was used as a measure of superoxide anions to determine the NADPH Oxidase activity [29]. In a microplate (Perkin-Elmer), freshly prepared hippocampus homogenates were incubated with DHE (10 µmol), salmon testes DNA (0.5 mg/ml, Sigma)in the presence or absence of β-NADPH (0.1 mM, Sigma) as substrate for 30 min at 37°C in a dark chamber. Ethidium-DNA fluorescence was measured at an excitation of 485±40 nm and an emission of 590±35 nm using fluorescence plate reader (Bio-Tek Instruments, Winooski, VT).

### PARP assay

Hippocampal homogenate equivalent to 50 µg of total protein was used for the determination of PARP-1 activity using HT Universal Colorimetric PARP-1 Assay Kit (Trevigen Inc, Md). The assay components were added according to manufacturer's instructions and reaction was terminated by adding 50 µl per well of 5% phosphoric acid and the absorbance was read at 450 nm.

### Caspase assay

The Caspase 3, 8 and 9 activity assays were performed using Caspase Fluorometric Assay kit (BioVision, USA). The assay is based on estimation of the intensity of fluorescence produced by cleavage of specific sequence (substrate) coupled with a fluorogenic compound, 7-amino-4-trifluoromethyl coumarin (AFC) which serves as a substrate. The tissue homogenate equivalent to 100 µg of total protein was diluted with 2× reaction buffer containing 10 mM DTT. Five microlitre of the respective substrate (Caspase-3: DEVD-AFC; Caspase-8: IETD-AFC; Caspase-9: LEHD-AFC) was added to the reaction mixture in micro-plate and incubated at 37°C for 1–2 h. The fluorescence intensity was measured using Bio-Tek FL600 fluorescence plate reader (Bio-Tek Instruments, Winooski, VT).

### Apoptotic neuronal death detection by TUNEL method:

To identify the apoptotic neuronal death in the sections, terminal deoxynucleotidyl transferase mediated biotinylated dUTP nick end-labeling (TUNEL) staining was performed with the Apo-BrdU-IHC *In Situ* DNA Fragmentation Assay Kit, following the manufacture's instruction (BioVision, Inc., USA) and developed with 3'3-diaminobenzidine in the presence of H_2_O_2_/urea tablet provided in the kit. After TUNEL reaction, the sections were counterstained with methyl green. The sections were mounted with glycerol/PBS (9∶1) and observed under 40× objectives on Nikon Eclipse E600 with Evolution VF camera. The captured images were analyzed with Image-Pro Plus version 5.1.2 software (Mediacybernetics).

### Statistical analysis

Data were analyzed using Graph Pad Prism 4.0 and Sigmaplot 9.0. All results were expressed as mean ± S.E.M for groups of 5–6 animals measured individually. Data were analyzed by one way analysis of variance (ANOVA) followed by post hoc comparison using Tukey's multiple comparison test. The p value <0.05 was considered statistically significant.

## Results

Hypobaric hypoxia is found to perturb the expression of a wide range of genes, but the homeostasis of free zinc released and its effect on inflammation and apoptosis has not been reported to the best of our knowledge. In the present study, we explored the effect of zinc chelation using Ca_2_EDTA on the expression of genes related to zinc homeostasis, inflammation and apoptosis in the hippocampus.

### Effect of Ca_2_EDTA on hypobaric hypoxia induced alteration in the expression of genes that regulate zinc homeostasis

**Figure 1:**
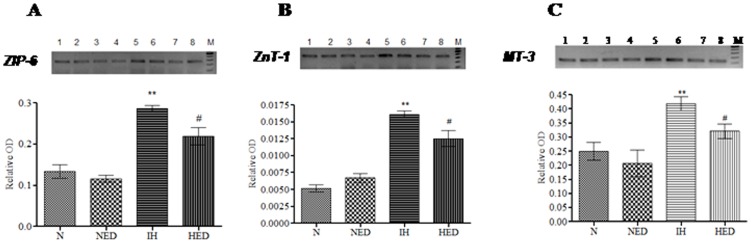
Effect of Ca_2_EDTA administration on the expression of genes that regulate zinc homeostasis in the hippocampus of animals after exposure to hypobaric hypoxia. Graph represents the relative OD of *ZIP-6* (335 bp) [A], *ZnT-1* (249 bp) [B] and *MT-3* (123 bp) [C] mRNAs expression in the hippocampus. The expression of all genes were normalized to the level of *GAPDH* mRNA and representative of three experiments in duplicate. ** p<0.01, # p<0.05. * compared hypoxia vs normoxia and normoxia treated with Ca_2_EDTA. # compared hypoxia treated Ca_2_EDTA vs hypoxia. N-normoxia, NED-normoxia treated with Ca_2_EDTA, IH-hypoxia and HED-hypoxia treated with Ca_2_EDTA. Lane 1,2-normoxia, Lane 3,4-normoxia treated with Ca_2_EDTA, Lane 5,6- hypoxia and Lane 7,8- hypoxia treated with Ca_2_EDTA. M indicate marker lane (A, B and C). *ZIP-6*: Forward-5′-AGCAGCCGACGATGTTGGAAGA-3′, Reverse: 5′- TGAAGGCAGCACCAATAGCAAG-3′ (335 bp); *ZnT-1*: Forward: 5′-TCGTGAATGCCTTGGTCTTTTA-3′, Reverse: 5′-GTTTGTAGAAGAATGAGAGCAGACT-3′ (249 bp); *MT-3*: Forward: 5′-CTGTCCTACTGGTGGTTCCTGC-3′, Reverse: 5′- GTCCTTGGCACACTTCTCACATC-3′ (123 bp).

The zinc homeostasis is maintained by transporters that are involved in the influx and efflux of zinc across the membrane and metallothioneins that buffer the intracellular free zinc. In the present study, we observed that hypobaric hypoxia significantly (*P*<0.01) up-regulated hippocampal mRNA expression of ZIP-6 as compared to normoxia and normoxia treated groups (one-way ANOVA: F_(3,17)_ = 80.07). The Ca_2_EDTA treated hypoxic animals showed significant (*P*<0.05) decrease in the mRNA expression of ZIP-6 as compared to hypoxic group (Fig 1A). Similarly the zinc transporter-1 (ZnT-1) mRNA expression was also significantly (*P*<0.01) increased in the hypoxic group as compared with normoxia and normoxia treated groups (one-way ANOVA: F_(3,20)_ = 66.40). The Ca_2_EDTA treatment significantly (*P*<0.05) decreased the mRNA expression of ZnT-1 as compared to hypoxic group (Fig 1B).

**Figure 2:**
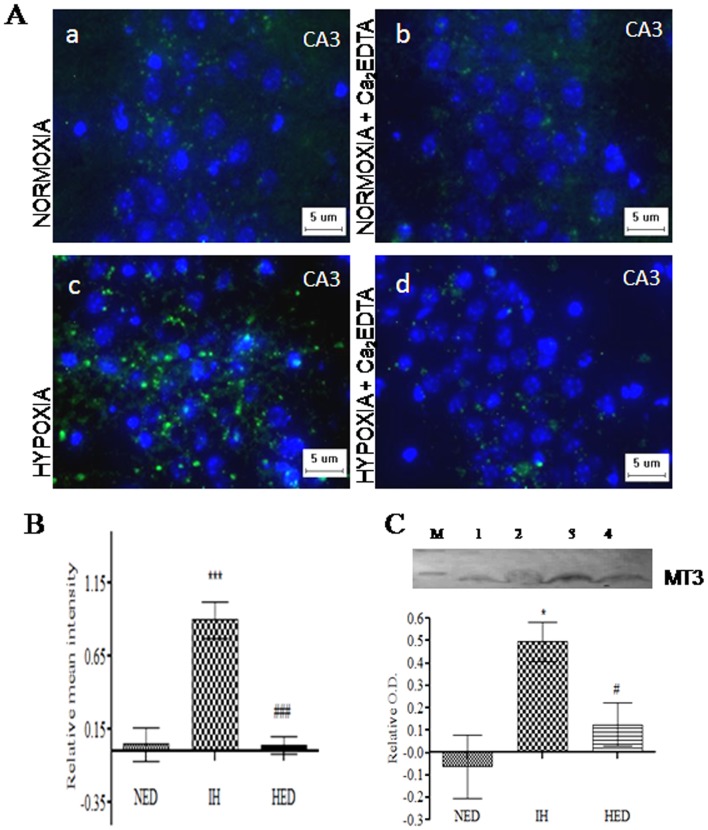
A. Photomicrograph shows the expression of MT-3 counter stained with Hoechst 33342 in CA3 hippocampal region of Normoxia (a), Normoxia treated with Ca_2_EDTA (b), Hypoxia (c), Hypoxia treated with Ca_2_EDTA (d) (Magnification ×400). **B**. Graph represents relative mean fluorescence intensity of MT-3 expression in hippocampal sections of CA3 region.**C**. Western blot analysis and quantification of MT-3 ***, ### indicate p<0.001. * compared hypoxia with normoxia treated with Ca_2_EDTA. # compared hypoxia treated Ca_2_EDTA vs hypoxia. NED-normoxia treated with Ca_2_EDTA, IH-hypoxia and HED-hypoxia treated with Ca_2_EDTA. Scale bar  = 5 µm.

The metallothionein-3 (MT-3) mRNA expression was significantly (*P*<0.01) increased in the hypoxic group as compared with normoxia, normoxia treated and hypoxic treated groups (one-way ANOVA: F_(3,20)_ = 17.45). The Ca_2_EDTA treated hypoxic animals showed significant (*P*<0.05) decrease in the mRNA expression of MT-3 (Fig 1C). Immunoblot analysis also showed that the hypobaric hypoxia significantly (*P*<0.05) increased the MT-3 protein expression as compared to normoxia and normoxia treated group (one-way ANOVA: F_(2,12)_ = 11.45). The observed increase in MT-3 protein expression was significantly (*P*<0.05) reduced by the zinc chelator (Fig 2 C). Immunofluorescence studies also substantiated the findings that hypobaric hypoxia significantly (*P*<0.001) increased MT-3 expression in the hippocampus CA3 region and zinc chelation significantly (*P*<0.001) reduced the MT-3 expression in the hippocampus (one-way ANOVA: F_(2,41)_ = 26.12) (Fig 2).

### Effect of Ca_2_EDTA on hypobaric hypoxia induced alteration in expression of HIF-1α

**Figure 3:**
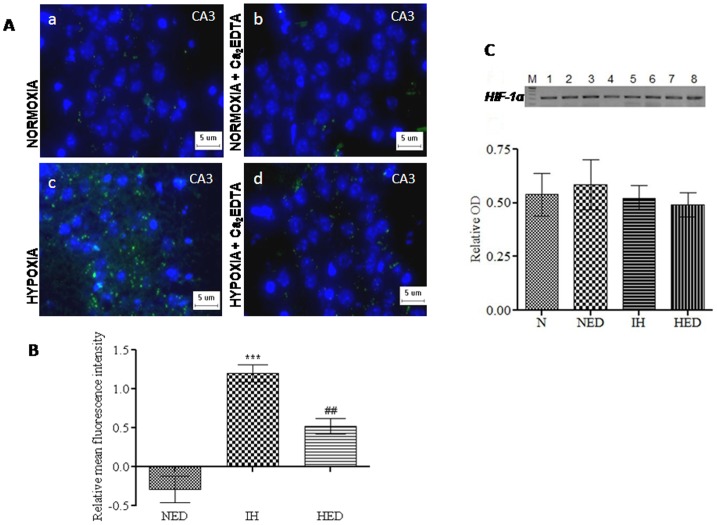
Effect of Ca_2_EDTA on hypobaric hypoxia induced alteration in expression of HIF-1α. Photomicrographs show the expression of HIF-1α counter stained with Hoechst 33342 (A) in CA3 hippocampal region of normoxia (a), normoxia treated with Ca_2_EDTA (b), hypoxia (c) and hypoxia treated with Ca_2_EDTA (d) (Magnification ×400). Graph represents relative mean fluorescence intensity of HIF-1α expression in hippocampal sections of CA3 region (B) and *HIF-1α* mRNA (216 bp) expression and relative OD (C) in hippocampus. *** indicate p<0.001;* compared hypoxia vs normoxia treated with Ca_2_EDTA. ## indicate p<0.01, # compared hypoxia hreated Ca_2_EDTA vs hypoxia. NED-Normoxia Treated with Ca_2_EDTA, IH-Hypoxia and HED-Hypoxia Treated with Ca_2_EDTA. Lane 1,2-Normoxia, Lane 3,4-Normoxia treated with Ca_2_EDTA, Lane 5,6- Hypoxia and Lane 7,8- Hypoxia treated with Ca_2_EDTA. M indicate Marker lane. *HIF-1α* – Forward primer: 5′ - AGAAACCTACCATCACTGCCACT- 3′, Reverse primer: 5′ – TGTTCTATGACTCTCTTTCCTGC - 3′ (216 bp). Scale bar  = 5 µm.

Brain, as a main consumer of energy, is particularly susceptible to hypoxia leading to neuronal dysfunction and cell death. HIF-1α plays a pivotal role in the signaling pathway of molecular and cellular adaptation to hypoxia. We observed that there was no significant change in the mRNA expression of HIF-1α in any of the groups employed (one-way ANOVA: F_(3,17)_ = 0.2386). But immunofluorescence studies demonstrated significantly (*P*<0.001) elevated levels of HIF-1α protein expression in the hypoxic group as compared with normoxia and normoxic Ca_2_EDTA treated animals (one-way ANOVA: F_(2,14)_ = 30.24). Ca_2_EDTA treatment significantly (*P*<0.01) reduced the HIF-1α expression in the hippocampus CA3 region of hypoxic animals Fig (3).

### Effect of Ca_2_EDTA on hypobaric hypoxia induced alteration in the expression of inflammatory mediators

**Figure 4:**
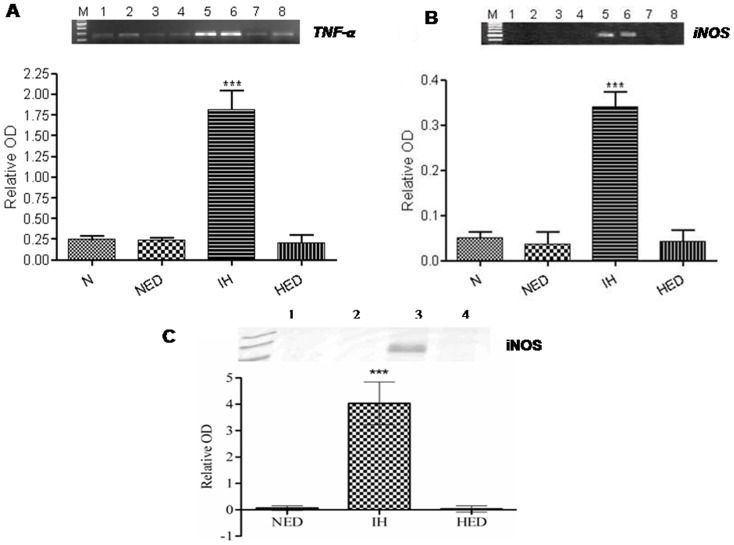
Effect of Ca_2_EDTA on hypobaric hypoxia induced alteration in the expression of inflammatory mediators. Graphs represent the relative OD of TNF-α mRNA (A); iNOS mRNA (B) and iNOS protein (C) expression in the hippocampus. ***, ### indicate p<0.001. * compared hypoxia vs normoxia and normoxia treated Ca_2_EDTA. # compared hypoxia treated Ca_2_EDTA vs hypoxia. N- Normoxia, NED-Normoxia treated with Ca_2_EDTA, IH-Hypoxia and HED-Hypoxia treated with Ca_2_EDTA. Lane 1,2-Normoxia, Lane 3,4-Normoxia treated with Ca_2_EDTA, Lane 5,6- Hypoxia and Lane 7,8- Hypoxia treated with Ca_2_EDTA. M indicate Marker lane. TNF-α: Forward Primer: 5′- CTGAGTTGGTCCCCCTTCT - 3′, Reverse primer: 5′ – CCGATGGGTTGTACCTTGT -3′ (240 bp); iNOS: Forward primer: 5′-ATGGACCAGTATAAGGCAAG-3′, Reverse primer: 5′-CTCTGGATGAGCCTATATTG-3′ (427 bp).

**Figure 5:**
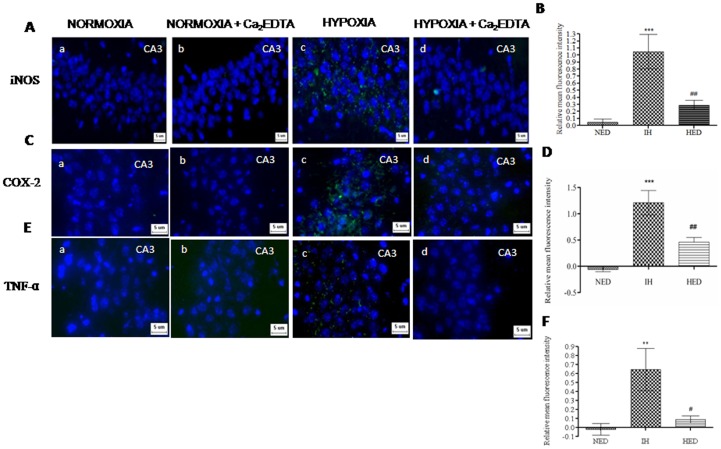
Effect of Ca_2_EDTA on hypobaric hypoxia induced alteration in the expression of inflammatory mediators. Photomicrographs show the expression of iNOS (A), COX-2 (C) and TNF-α (E), counter stained with Hoechst 33342 on CA3 hippocampal regions of normoxia (a), normoxia treated with Ca_2_EDTA (b), hypoxia (c), hypoxia treated with Ca_2_EDTA (d) (Magnification ×400). Graph represents relative mean fluorescence intensity of iNOS (B), COX-2 (D) and TNF-α (F) expression in hippocampal sections of CA3 region. *** indicate p<0.001, **, ## indicate p<0.01, # indicate p<0.05. * compared hypoxia vs normoxia treated Ca_2_EDTA. # compared hypoxia treated Ca_2_EDTA vs hypoxia. NED-normoxia treated with Ca_2_EDTA, IH-hypoxia and HED-hypoxia treated with Ca_2_EDTA. Scale bar  = 5 µm.

Inflammatory neurodegeneration is implicated in diseases like Alzheimer's, Parkinson's, Multiple sclerosis, etc. In the present study, we investigated the effect of Ca_2_EDTA on the status of inflammatory mediators expression in the hippocampus. We observed that normoxia, normoxia treatment and hypoxia treatment did not show any detectable mRNA expression of iNOS. Hypobaric hypoxia resulted in significantly (*P*<0.001) increased iNOS mRNA expression in the hippocampus, which was significantly (*P*<0.001) reduced by Ca_2_EDTA treatment (one-way ANOVA: F_(3,20)_ = 88.25) (Fig 4B). Similarly, immunoblot (one-way ANOVA: F_(2,15)_ = 34.65) (Fig 4C) as well as immunofluorescence (one-way ANOVA: F_(2,28)_ = 16.29) (Fig 5A & B) studies showed significantly (*P*<0.001) elevated iNOS protein expression in hipocampii of the hypoxic group. Administration of zinc chelator to hypoxic animals significantly (*P*<0.01) reduced the iNOS expression as compared to untreated hypoxic animals. Further, hypoxia significantly (*P*<0.001) increasedTNF α mRNA level (one-way: ANOVA F_(3,20)_ = 71.52) (Fig 4A) and protein expression (one-way ANOVA: F_(2,19)_ = 9.334) (Fig 5E & F) as compared to normoxia and normoxia treated group. Ca_2_EDTA treatment to hypoxic animals significantly (*P*<0.05) attenuated the elevation in TNF-α expression as compared to hypoxic group. Immunofluorescence results revealed that COX-2 expression was significantly (*P*<0.001) elevated in the hypoxic group as compared with normoxia and normoxia Ca_2_EDTA treated group (one-way ANOVA: F_(2,15)_ = 20.06). The Ca_2_EDTA treated hypoxic group significantly (*P*<0.01) reduced COX-2 expression as compared to hypoxic group (Fig 5C& D).

### Effect of Ca_2_EDTA on hypobaric hypoxia induced alteration in NADPH Oxidase activity

**Figure 6:**
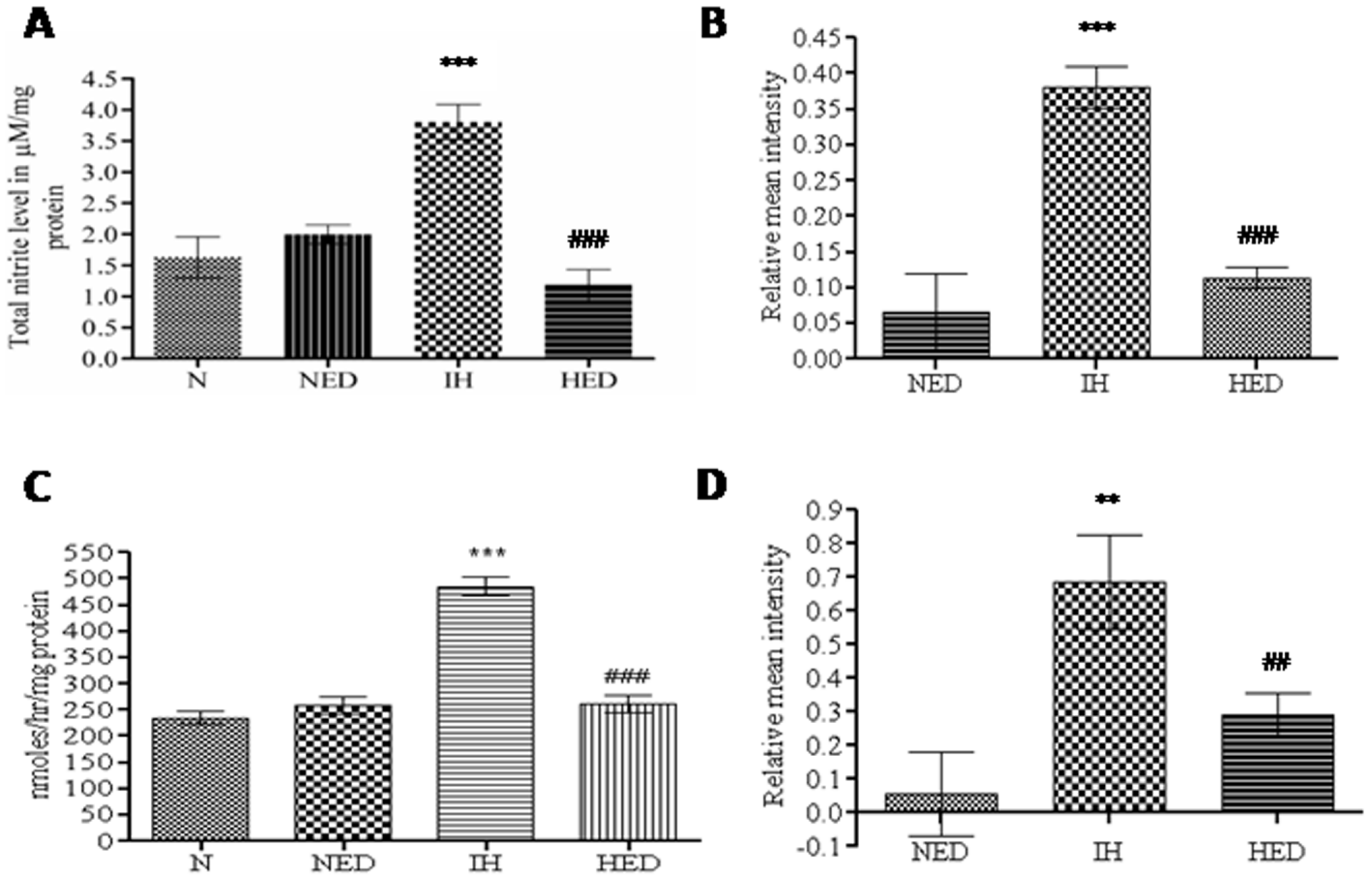
Effect of Ca_2_EDTA on nitrite content, NADPH oxidase activity, LPO level, and PARPactivity. Graph represents the total nitrite level (A), NADPH Oxidase activity (B), lipid peroxidation (C) and PARP activity assay (D) in the hippocampal tissue homogenate. ***, ### indicate p<0.001, **, ## indicate p<0.01. * compared hypoxia vs normoxia and normoxia treated Ca_2_EDTA, # compared hypoxia treated Ca_2_EDTA vs hypoxia. N-Normoxia, NED-Normoxia Treated with Ca_2_EDTA, IH-Hypoxia and HED-Hypoxia Treated with Ca_2_EDTA.

NADPH oxidase, widely expressed in various tissues including neurons and microglia, catalyses the conversion of oxygen to super oxide anions (^-•^O_2_). The ^-•^O_2_ serves as precursor of a complex array of highly reactive potent oxidants like ^•^OH and ^–^ONOO, thereby further accelerating the neuronal damage. Its role in hypobaric hypoxia still remains to be evaluated. Herein, we observed that NADPH oxidase activity was significantly (*P*<0.001) increased in the hypoxic group as compared to normoxia and normoxic treated group (one-way ANOVA: F_(2,12)_ = 21.10). Treatment with zinc chelator significantly (*P*<0.001) reduced NADPH oxidase activity in the hipocampii of hypoxic animals as compared with untreated hypoxic animals (Fig 6B).

### Effect of Ca_2_EDTA on hypobaric hypoxia induced lipid peroxidation and NO levels

Hippocampii of hypoxic animals showed significant (*P*<0.001) elevation in malondialdehyde levels than normoxia and normoxia treated animals (one-way ANOVA: F_(3,20)_ = 104.4). Ca_2_EDTA treatment significantly (*P*<0.001) attenuated the hypoxia induced increase in MDA levels (Fig 6C). NO is known to release free zinc from the metallothioneins and its involvement in neuronal injury is well documented. In the present study, we made an effort to evaluate the effect of Ca_2_EDTA on hypobaric hypoxia induced elevation in nitric oxide. Hipocampii of hypoxic animals showed a significant (*P*<0.001) elevation in NO level, which was higher than normoxia and normoxic treated groups (one-way ANOVA: F_(3,20)_ = 64.45). However, treatment with zinc chelator significantly (*P*<0.001) decreased the hypoxia induced increase in nitric oxide level (Fig 6A).

### Effect of Ca_2_EDTA on hypobaric hypoxia induced alteration in PARP activity

Poly(ADP Ribose)Polymerase (PARP) functions as a DNA repair enzyme, but its over-activation results in energy failure and cellular damage. In this study, hypobaric hypoxia showed significantly (*P*<0.001) increased PARP activity and chelation of free zinc with Ca_2_EDTA resulted in significant (*P*<0.05) reduction of PARP activity when compared with hypoxic group (one-way ANOVA: F_(2,17)_ = 21.23). Further, it was noticed that the hypoxia treated group showed insignificantly elevated PARP activity as compared with normoxia and normoxic treated group (Fig 6D).

### Effect of Ca_2_EDTA on hypobaric hypoxia induced alteration in apoptotic mediators

**Figure 7:**
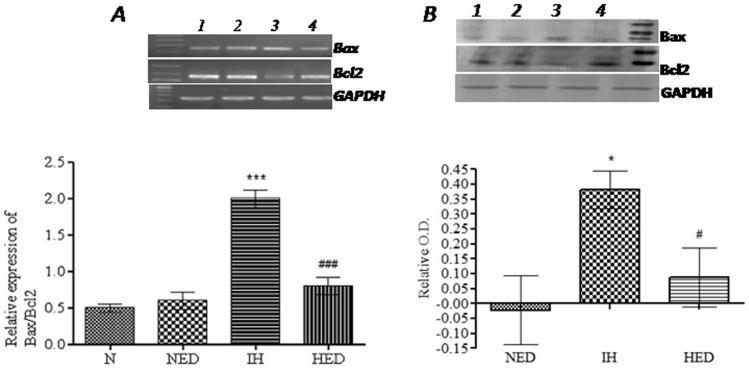
Effect of free chelatable zinc on hypobaric hypoxia induced alteration in Bax/Bcl-2 expression. Graph represents the relative mRNA expression of Bax/Bcl-2 in the hippocampus (A). Lane 1-Normoxia, Lane 2-Normoxia treated with Ca_2_EDTA, Lane 3- Hypoxia and Lane 4- Hypoxia treated with Ca_2_EDTA. Graph (B) represents the relative protein expression of Bax/Bcl-2 in the hippocampus. Lane 1-Normoxia, Lane 2-Normoxia treated with Ca_2_EDTA, Lane 3- Hypoxia and Lane 4- Hypoxia treated with Ca_2_EDTA. *, # indicate p<0.05. ***, ### indicate p<0.001. * compared hypoxia vs normoxia and normoxia treated Ca_2_EDTA. # compared hypoxia treated Ca_2_EDTA vs hypoxia. N- Normoxia, NED-Normoxia Treated with Ca_2_EDTA, IH-Hypoxia and HED-Hypoxia Treated with Ca_2_EDTA. Bcl-2: Forward -5′-CTGGCATCTTCTCCTTCCAG-3′; Reverse - 5′-GACGGTAGCGACGAGAGAAG-3′ (183 bp); Bax: Forward: 5′-TGAAGACAGGGGCCTTTTTG-3′; Reverse: 5′-AATTCGCCGGAGACACTCG-3′ (139 bp).

**Figure 8:**
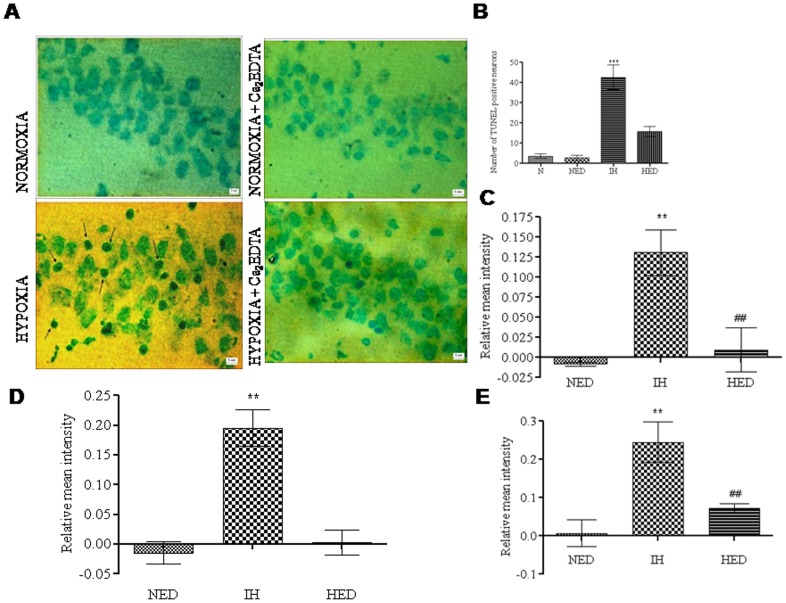
Effect of free chelatable zinc on hypobaric hypoxia induced apoptosis. A. Photomicrographs of TUNEL stained hippocampus CA3 region (A) of normoxia (a), normoxia treated with Ca_2_EDTA (b), hypoxia (c), and hypoxia treated with Ca_2_EDTA (d) (Magnification 400X). Arrow head shows TUNEL positive neurons. Graph (B) represents the number of TUNEL positive neurons from the sections of hippocampus CA3 region, caspase 9 (C), caspase 8 (D) and caspase 3 (E) activities. *** indicate p<0.01, **, ## indicate p<0.01. * compared hypoxia vs normoxia and normoxia treated Ca_2_EDTA and hypoxia treated with Ca_2_EDTA. # compared hypoxia treated Ca_2_EDTA vs hypoxia. N- Normoxia, NED-Normoxia Treated with Ca_2_EDTA, IH-Hypoxia and HED-Hypoxia Treated with Ca_2_EDTA. Scale bar  = 5 µm.

We observed that hypobaric hypoxia showed significant (mRNA expression *P*<0.001; protein expression *P*<0.05) increase in the relative Bax/Bcl-2 expression as compared with the normoxic and normoxic treated groups in the hippocampus (one-way ANOVA: F_(3,20)_ = 75.45; F_(2,23)_ = 12.86, respectively). Ca_2_EDTA treated group showed significant reduction (mRNA expression *P*<0.001; protein expression *P*<0.05) in the relative Bax/Bcl-2 expression when compared with the hypoxic group (Fig 7A&B). Further, caspase family of proteases play a central role in apoptosis. We observed that caspase 9 & 8 activities were significantly (*P*<0.01) increased when compared with normoxia and normoxia treated groups. Treatment with zinc chelator significantly (*P*<0.01) reduced both caspase 9 & 8 activities as compared with hypoxic group (one-way ANOVA: F_(2,16)_ = 9.876; F_(2,17)_ = 54.62, respectively) (Fig 8C & D). In addition, the activity of effector protease, caspase 3 was significantly (*P*<0.01) increased in hippocampii of hypoxic group when compared with normoxia and normoxia treated groups (one-way ANOVA: F_(2,21)_ = 25.05). Treatment with zinc chelator significantly (*P*<0.01) reduced the caspase 3 activity as compared with hypoxic group (Fig 8E).

### Effect of Ca_2_EDTA on hypobaric hypoxia induced apoptosis (TUNEL assay)

The number of apoptotic neurons in the hippocampus CA3 region was significantly (*P*<0.001) higher in the hypoxic group as compared with the normoxic group. Further, treatment with zinc chelator showed significant (*P*<0.01) protection against apoptosis in the hippocampus during hypobaric hypoxia (one-way ANOVA: F_(3,24)_ = 30.05). There was significant (*P*<0.05) difference in the number of apoptotic neurons between hypoxic treated and the normoxic groups (Fig 8A & B).

## Discussion

Molecular pathways underlying hypoxia induced neurotoxicity and memory dysfunction are multifaceted involving oxidative stress, mitochondrial dysfunction and activation of apoptotic cascades [1], [30], [31]. Zinc is manifested in neurodegenerative conditions like ischemic hypoxia in the hippocampal region, a vital region involved in the memory processes [32]. The zinc homeostasis involves a variety of zinc transporters and zinc buffering proteins. In the present study, we observed that hypobaric hypoxia upregulated the expression of ZIP-6 and ZnT-1, which act by transporting zinc from extracellular space to cytoplasm and *vice-versa*, respectively. Ca_2_EDTA could partially attenuate the hypoxia induced alteration in the expression of both ZnT-1 and ZIP-6. Further, hypobaric hypoxia resulted in the elevation of MT-3, a brain specific intracellular zinc buffering protein and was significantly attenuated by the zinc chelator. These observations and the results from our previous study suggest that increased levels of transporters and zinc buffering protein (MT-3) under hypoxic condition may lead to higher intracellular levels of free zinc particularly in the pyramidal neurons of hypoxia susceptible CA-3 region resulting in neuronal damage and memory impairment [20].

### Zinc chelation modulates the oxidative stress and pro-inflammatory gene expression

Metallothioneins are highly efficient free radical scavengers, known to be involved in cytoprotection [33] and are associated with oxidative stress and inflammation [34], [35]. Our results showed elevated levels of malondialdehyde, a marker of lipid peroxidation suggesting the existence of oxidative stress during hypobaric hypoxia. Further, the mRNA and protein expression of iNOS, an inflammatory mediator, was also up-regulated in the hippocampus of hypoxic animals. In addition, we observed increased NO levels in the hippocampii of the hypoxic group almost twice the level of normoxic group. This inflammatory response was associated with up-regulation of COX-2 and TNF-α expression in the hypoxic animals. Ca_2_EDTA showed neuroprotective effect against hypobaric hypoxia by reducing the oxidative stress and regulation of inflammatory genes in the hippocampus after exposure to hypobaric hypoxia. These results suggest that the free zinc released during hypoxia might play an instrumental role in hypoxia mediated pathophysiological alterations in the hippocampus. Our previous findings and other reports suggest that iNOS mediated NO release is associated with memory dysfunction and neuronal damage [3], [36]. Moreover, it has been reported that iNOS plays a detrimental role in the behaviour of animals following global cerebral ischemia [36], intermittent hypoxia [37], intracerebroventricular administration of β-amyloid [38] and lypopolysaccharides (LPS) [39]. Earlier reports also suggested the involvement of nitric oxide in pathophysiology of neurological disorders under hypoxic condition [3], [40], [41], [42], which is endogenously produced by iNOS [43]. Our present study has revealed an interesting fact that free zinc released during hypobaric hypoxia might be an essential factor for the consequences exerted by iNOS. This might be due to the effect of free zinc on the alteration of iNOS expression at transcriptional level. Moreover, iNOS dimers are stabilized with zinc metal at the centre, which is necessary for the synthesis of NO [44]. It is known that the expression of iNOS is regulated by HIF-1α [45], a key regulator of cellular and systemic responses to hypoxia. Here in, we observed that mRNA expression of HIF-1α was not altered in any of the groups but the protein expression was found to be elevated in the hypoxic animals. Evidences suggest that during hypoxia, increasing ROS might contribute to the stabilization of HIF-1α [9], [46], [47] and subsequently translocated to the nucleus where it binds constitutively expressed HIF1-β, forming HIF-1. HIF-1 binds hypoxia response elements on numerous genes encoding proteins such as erythropoietin, vascular endothelial growth factor (VEGF) and numerous glycolytic enzymes necessary for responding to hypoxic stress [6], [7], [8], thereby influencing metabolic adaptation, cell survival and apoptosis [48]. In the present study, Ca_2_EDTA reduced the elevated expression of HIF-1α at protein level on exposure to hypobaric hypoxia, which suggests that free zinc released during hypobaric hypoxia might contribute to the stabilization of HIF-1α [49]. The relative contribution of ROS to stabilize HIF-1α suggested by others has been substantiated in our study. Hence, we further investigated whether zinc chelator alters the ROS generation in the hippocampus of hypoxic animals. NADPH oxidase, a major source of ROS in biological system including mouse hippocampal neurons [50] was found to have increased activity in the hypoxic animals. The hypoxic animals treated with zinc chelator markedly attenuated the hypoxia induced elevated NADPH oxidase activity in hippocampus and hence the reduction in reactive oxygen species. Similar to our results, NADPH oxidase activation has been reported in rats and gerbils following cerebral ischemia [51], [52]. Further, toxic concentration of zinc is known to damage the cortical neurons via NADPH mediated superoxide release [53]. Moreover, free radicals mediated lipid peroxidation and inflammation associated with microglia activation in the hippocampus of ischemic animals was attenuated by NADPH oxidase inhibitor [52]. These reports suggest that hypobaric hypoxia induced zinc release mediates ROS production, which is an essential component for the activation and stabilization of HIF-1α [54], [55].

### Zinc chelation attenuated the hypobaric hypoxia induced apoptosis in hippocampus

In the present study, we observed that hypobaric hypoxia resulted in generation of nitric oxide as well as superoxide, which are known to facilitate the production of a peroxynitrite. Peroxynitrite is a highly potent oxidant that has a destructive effect on DNA. PARP, an enzyme known to maintain the integrity of DNA has been implicated in neurodegenerative disorders and PARP inhibitors were found to be protective against neuronal injury [27], [56]. We observed that hypoxia resulted in activation of PARP in the hippocampus, which was effectively suppressed by the zinc chelator. Current findings suggest that free zinc released during hypobaric hypoxia modulates PARP activity resulting in HIF-1α accumulation in the presence of nitrative and oxidative stress [57]. The involvement of hypobaric hypoxia induced zinc release in mediating apoptotic neuronal death was evident, as the zinc chelator treatment to hypoxic animals resulted in reduced programmed cell death in CA3 region of hippocampus, as revealed from TUNEL assay. The results of TUNEL assay were substantiated by caspase activity assays. The experimental results showed increased activity of caspase 9 and 3, the markers for the initiation and execution of apoptosis, respectively. Zinc chelation showed significant reduction in the activity of both caspase 9 and 3 in the hippocampal homogenate of hypoxic animals. It has been recently reported that zinc modulates the expression of caspase 9&3 in the hippocampus following transient global ischemia in gerbils [32], which strongly support our present findings. Further, it is known that Bcl-2 family proteins play a prominent anti-apoptotic role by acting upstream of caspase activation and PARP has a modulatory effect on Bcl-2 expression [58]. In our present study, we observed that the Bax/Bcl-2 ratio was increased in the hypoxic animals thereby suggesting a bax mediated caspase activation and neuronal death. Reduction in Bax/Bcl-2 ratio and neuronal apoptosis, as observed in zinc chelator treated group substantiates the involvement of zinc in neurotoxicity during hypoxic condition. These results suggest that free zinc is associated with oxidative stress mediated DNA damage, leading to the activation PARP and further resulting in Bax mediated cellular damage. However, it is also possible that free zinc might have a direct regulatory role in the expression of Bcl-2 and/or Bax, which needs further investigation. Current findings suggest that hypobaric hypoxia induces intrinsic apoptotic mechanisms and are selectively localized to particular cellular populations within the hippocampus, especially CA3 region. Further, upregulation of TNF-α expression after exposure to hypobaric hypoxia prompted us to study whether extrinsic factors are also involved. Interestingly, we observed that caspase 8 activity was also increased in hippocampiis of hypoxic animals, which was attenuated by zinc chelator. All these data suggest that free zinc released during hypobaric hypoxia might be involved in the intrinsic as well as extrinsic pathway of the apoptotic mechanism. Similar to our observations in hippocampus, it has been reported that cerebral hypoxia resulted in increased activity of caspase-9 and caspase-3 in the cerebral cortex of newborn piglets [59], [60]. Moreover, hypoxia resulted in oxidative stress mediated increase in pro-apoptotic proteins such as Bax, APAF-1 with subsequent down-regulation of anti-apoptotic Bcl-2 and activation of caspase-3 [61] leading to neuronal apoptosis.

## Conclusions

**Figure 9:**
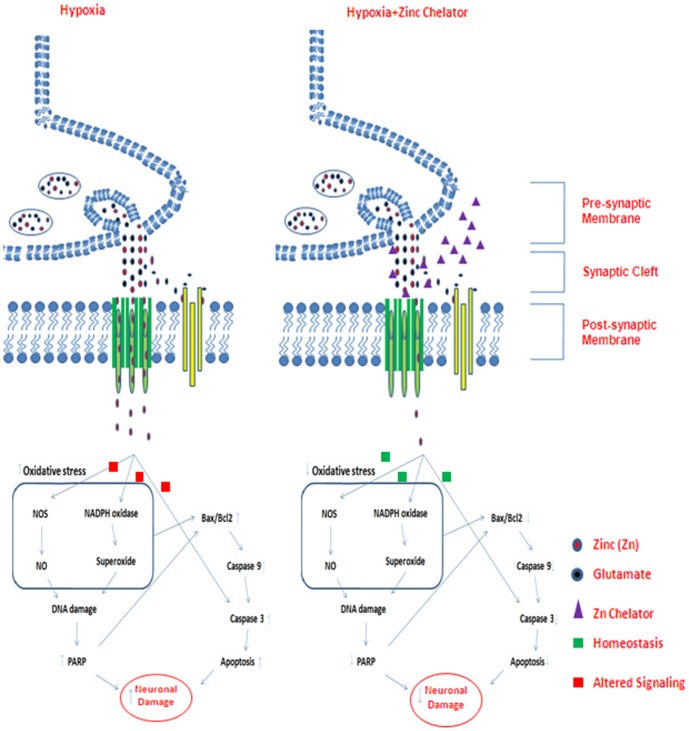
Schematic illustration of the role of free chelatable zinc during hypoxic conditions. Zinc chelator alters the accumulation of free zinc in the hippocampus thus, reduces oxidative stress and inhibits PARP activation. Oxidative stress and PARP collectively modulate Bcl-2 expression resulting in Bax mediated activation of caspases leading to apoptotic neurodegeneration during hypoxic stress which is reduced significantly by the treatment zinc chelator.

It can be concluded that zinc chelator could effectively attenuate the hypobaric hypoxia induced superoxide generation via NADPH oxidase, leading to a destabilization effect on HIF-1α and resultant maintenance of low expression of genes such as iNOS, MT-3, TNFα, etc. Apart from protection against neuronal inflammation, the zinc chelator could also influence the apoptotic neuronal damage induced by hypobaric hypoxia in the hippocampus CA3 region of brain (Fig 9). Altogether, the results of the present study enhance our understanding of the role of free chelatable zinc in the pathophysiology of neuronal inflammation and apoptosis, implying that chelation of free zinc may be useful in the therapy of neuropathological conditions such as hypobaric hypoxia.
